# Association of the age and bodyweight at first calving with the reproductive and productive performance in one herd of Holstein dairy heifers in Japan

**DOI:** 10.1002/vro2.44

**Published:** 2022-09-15

**Authors:** Hiromi Kusaka, Takeshi Yamazaki, Minoru Sakaguchi

**Affiliations:** ^1^ Laboratory of Theriogenology School of Veterinary Medicine Kitasato University Towada Aomori Japan; ^2^ Hokkaido Agricultural Research Center National Agriculture and Food Research Organization (NARO) Sapporo Japan

## Abstract

**Background:**

Reducing the age at first calving (AFC) in dairy heifers may decrease replacement costs, while the acceleration of body growth could affect milk productivity. A lower bodyweight (BW) at first calving may increase calving problems and compromise the subsequent reproductive performance.

**Materials and methods:**

This retrospective study aimed to investigate the effect of AFC and BW prior to calving on milk productivity, the incidence of calving problems (difficult calving and stillbirth) and reproductive performance during the first lactation. Multivariate analysis was conducted using a total of 203 calving records from 1999 to 2012 for one herd of Holstein heifers. The AFC was categorised as young, moderate, old and very old (<22.5, 22.5 to <24.0, 24.0 to <25.5, ≥25.5 months) and the heifer BW before first calving was grouped into low, moderate, high and very high (≤625, 626–654, 655–683, ≥684 kg), respectively.

**Results:**

The incidence of difficult calving and the prevalence of stillbirth were significantly higher in the animals with low BW compared with the heifers with moderate and high BW. Even so, there was no adverse impact on reproductive performance. There was a significant association between the lifetime daily milk yield and AFC; the highest mean value for yield was recorded for the heifers in the young AFC group, which was significantly different from heifers in the moderate and old age groups.

**Conclusions:**

In this experimental herd, a reduction in AFC could increase the profitability during the first lactation.

## INTRODUCTION

With the progress of genetic and feeding improvements to increase milk yield, replacement heifers can reach an appropriate bodyweight (BW) faster prior to first calving; consequently, the reduction of age at first calving (AFC) can decrease replacement costs.^1^ Furthermore, reductions in AFC may contribute to decreasing greenhouse gas emissions.^2^ As reviewed, the AFC averaged 26.0 months in the USA, 26.4 months in the UK, 28.8 months in Australia, 29.3 months in China and 31.0 months in Kenya.^3^ In Japan, it has been reported, in the past decade, that approximately 38% of Holstein heifers had their first calving at 24–26 months, with 24% calved at more than 27 months.^4^ The considerable variations in first calving age observed both within and between farms may be related to differences in age at first breeding due to farm management and/or growth rate, or it may be primarily related to heifer fertility.^5^ For modern dairy replacement heifers, adequate AFC and BW at the first calving have been proposed. The average AFC in Holsteins heifers has been recommended to be less than or equal to 24 months of age with BW greater than 560 kg at calving.^6^, ^7^ Other studies have implied that the AFC could be reduced further; for example, to less than 23 months, as long as the heifer has sufficient BW.^8^, ^9^

To reduce the AFC and maximise milk productivity and reproductive performance, heifers must acquire mature frame size and adequate BW by the time of the first calving. Most mammary gland development occurs before the first calving; therefore, it is important for heifers to maintain sufficient body growth during the prepubertal period. However, it has been reported that there is a deleterious effect of prepubertal rapid weight gain on mammogenesis when accompanied by excess body fat deposition. This negative effect of early calving on milk productivity has been reported in several studies.^9^, ^10^, ^11^ Another study reported a deleterious effect of prepubertal rapid weight gain on mammogenesis when accompanied by excess body fat deposition; however, this effect was not associated with a decline in subsequent milk production.^12^ Other studies have reported that a younger AFC in dairy heifers does not reduce or may even increase the first lactation yield, providing the animals are sufficiently well grown; namely, BW at first calving reaches around 600 kg for Holstein heifers.^5^, ^8^, ^13^, ^14^

In addition, a younger calving age can potentially impair subsequent reproductive performance. The most common cause of difficulty in calving is fetal–maternal disproportion: a calf that is too big and/or a pelvis that is too small. Therefore, replacement heifers must rapidly acquire a mature frame size and sufficient BW prior to their first calving. It has been suggested that inadequate skeletal maturity can be a problem during the first calving, especially if the AFC is less than 24 months,^15^ while other studies have suggested that the AFC could be reduced to 22 months without an increase in the frequency of problems at parturition.^16^, ^17^

There is a lot of interest in the reduction of the AFC; however, it is not easy to assess the effect of AFC on milk productivity and its association with calving problems and further reproductive performance due to various confounders. In particular, the effect of AFC and BW are difficult to separate because their relationship is highly correlated. Several prospective studies have reported the effect of AFC on milk productivity and reproductive performance, controlling the body growth rate until the first calving,^8^, ^9^, ^11^ while many retrospective studies, using large‐scale datasets, could not evaluate the association between the AFC and BW.^18^, ^19^, ^20^, ^21^, ^22^ Furthermore, less information is available on the productive and reproductive status of heifers at different AFC or BW under the same management conditions.^8^, ^9^, ^23^

A retrospective analysis of the calving records of 267 Holstein heifers in the Hokkaido Agricultural Research Centre, National Agriculture and Food Research Organization (NARO) (Sapporo, Japan) from 1979 to 1997 reported an association between AFC and BW at first calving.^24^ The results indicated that BW at first calving and milk yield level had significantly increased for 20 years; thus, early initiation of first breeding had no detrimental impact on the first lactation and led to better reproductive performance. A secondary prospective study, using the same experimental herd, reported that a reduction of AFC from 25 to 22 months was not associated with an increase in calving difficulty if the BW of heifers reached 600 kg at calving. An AFC of 22 months had no adverse effects on cumulative milk yields up to the third lactation or on reproductive performance.^8^ However, further investigation was required because the study sample size was limited.

The objective of the present study was to investigate the association of AFC and BW prior to calving with milk productivity, the incidence of calving problems (difficult calving and stillbirth) and, further, reproductive performance during the first lactation. We conducted a retrospective analysis using the calving records of the NARO from 1999 to 2012, considering some confounders, including delivered calf BW, genetic change and calving season.

## MATERIALS AND METHODS

### Animals and feed management

This study was conducted retrospectively using calving records from 1999 to 2012 for heifers in the Hokkaido Agricultural Research Centre. The heifers were managed as previously reported.^8^ The animals were reared using a feeding regimen to meet the maintenance, growth and lactation requirements and followed the Japanese Feeding Standard for Dairy Cattle (Agriculture, Forestry and Fisheries Research Council Secretariat, 1999; see https://jlia.lin.gr.jp/info/archives1627/. Accessed 22 Aug 2022). Whole milk (for the first 12 weeks) combined with calf starter grower (for the first 15 weeks) were fed up with a maximum of 5.0 and 1.2 kg/day, respectively. The calves were housed individually in calf stalls and, after weaning, they were moved to a tie‐stall barn and fed concentrate with grass silage until 12 months of age. The weaned heifer calves were pastured for 6 h/day during the summer period (May–October) and fed hay ad libitum in the paddock for 6 h/day during the winter period (November–April). From the age of 12 months to 1 month before parturition, the heifers were raised on pasture supplemented with alfalfa silage and grass hay packaged in rolls during summer; or fed corn silage and grass hay at a tie‐stall barn during winter.

Heifers confirmed to be pregnant in winter were fed rolled alfalfa silage and rolled grass hay ad libitum in an open paddock. From 1 month before calving to the end of each lactation period, the heifers were fed concentrate and grass silage with grass hay ad libitum. Lactating cows were housed in a free‐stall barn and milked twice daily (09:00 and 19:00 h). During summer, the lactating cows were pastured for 3–4 h/day; the amount of feed was reduced to meet the nutritional requirements during this period. Pregnant dry cows were pastured daily during summer.

### Reproductive management

Artificial insemination (AI) was initiated at the first oestrous for each heifer weighing more than or equal to 350 kg. All inseminations were performed using frozen–thawed semen from bulls in which normal fertility was confirmed. Conception was confirmed by the detection of a fetal heartbeat using ultrasonography at 35–40 days after each insemination. Postpartum cows were observed twice daily for at least 30 min before milking and those observed exhibiting standing oestrous or mounting activity accompanied by other signs, such as vaginal mucous discharge and swelling of the vulva, were considered to be in oestrous. Cows in oestrous were inseminated only after a minimum 45‐day voluntary waiting period post‐calving.

### Data collection

A total of 203 calving records of heifers from 1999 to 2012 were used. The age at the first calving was recorded and BW was measured before and within 1 week of parturition. Each delivered calf was weighed within 24 h after birth. The average daily liveweight gain (ADG) of the heifers from birth to the first calving was calculated by subtracting their calf weight from the BW before the calving and divided by the AFC. Calving difficulty was scored as 1 (no assistance), 2 (slightly assisted), 3 (assisted by two or three persons), 4 (assisted by four or more persons) and 5 (needed surgical treatment or death of dam) as previously reported.^25^ Twin births and stillbirths were recorded.

The following outcomes of reproductive performance were collected after the first calving: submission rate (%) for AI, the interval from calving to the first service, first service conception rate, final conception rate, number of services per conception and the number of days open.

The number of days in milk was recorded during the first lactation period. To assess milk yield productivity, 40 cows were excluded because of a short lactation period (<200 days in milk). Although there was limited information about the reasons for the short lactation; it was possible that the cows suffered from mastitis or hoof disease. For a total of 163 cows, the 305‐day milk yield was estimated and the age at the end of the first lactation was calculated by adding 305 days to the AFC. As an indicator of total productivity, the average daily milk yield from birth to the end of the first lactation (lifetime daily milk yield) was calculated by dividing the 305‐day milk yield by the age at the end of the first lactation.

### Statistical analysis

All statistical analyses were performed using JMP statistical software (JMP Pro Statistics and Graphics Guide, version 16.1.0; SAS Institute, Cary, NC, USA). Statistical significance was set at *p* < 0.05. Continuous variables are presented as means ± standard deviation.

A total of 203 heifers were categorised retrospectively according to the AFC as young (<22.5 months, *n* = 25), moderate (22.5 to <24.0 months, *n* = 80), old (24.0 to <25.5 months, *n* = 68) and very old (≥25.5 months, *n* = 30). The categories were based on a previous study of the same herd, which had proposed that for a heifer with AFC less than 22.0 months, there was no adverse effect on subsequent fertility and milk productivity.^8^ Heifer BW before first calving was grouped into four categories including low (≤625 kg, *n* = 49), moderate (626–654 kg, *n* = 52), high (655–683 kg, *n* = 50) and very high (≥684 kg, *n* = 52); these were based on the 25th percentile, the median and the 75th percentile of the BW data. The delivered calf BW was grouped into three categories including low (21.0–38.0 kg, *n* = 51), moderate (38.5–44.0 kg, *n* = 101) and high (44.5–75.5 kg, *n* = 51); these were based on the 25th and 75th percentiles of the BW data. The BW was totaled for twins. Descriptive analysis was conducted for AFC and BW.

To consider genetic changes (provided through repeated breeding and improvement) and seasonal effects, the years and season of the first calving were grouped into four categories. For years, period 1 (1999–2002, *n* = 46), period 2 (2003–2006, *n* = 68), period 3 (2007–2009, *n* = 53) and period 4 (2010–2012, *n* = 36); for seasons, Spring (April–June, *n* = 54), Summer (July–September, *n* = 36), Autumn (October–December, *n* = 46) and Winter (January–March, *n* = 67). The AFC, BW (heifer and calf), year‐period and season were used as effects when performing the analysis.

### Incidence of difficult calving and prevalence of stillbirth

Multivariate logistic regression analysis was performed to evaluate the effect of AFC, BW (heifer and calf), year‐period and season, on the outcome variables of the calving problem. The incidence of difficult calving and the prevalence of stillbirth were the outcome variables. A likelihood ratio test was performed. If a significant difference was detected in the categories of each effect, a univariate logistic regression analysis was performed between the calving problem and the effect; odds ratios and 95% confidence intervals (CIs) were calculated.

### Reproductive performance and calf production

Multivariate analysis using a generalised linear model was performed to evaluate the association of AFC, BW (heifer and calf), year‐period and season, with the outcome variables of reproductive performance and calf production (calf number and weight per heifer). The interval from calving to the first service, the number of days open, calf production per heifer and BW for delivered calves, were the outcome variables. If a significant difference was detected, multiple comparisons were performed using a Tukey's honestly significant difference (HSD) test to analyse the differences between the categories for each effect.

### Lactation performance

Multivariate logistic regression analysis was performed to evaluate the association of AFC, heifer BW, year‐period and season, with the outcome variables of the short lactation. The frequency of heifers having less than 200 days in milk was the outcome variable. If a significant difference was detected in the categories of each effect, a univariate logistic regression analysis was performed between the calving problem and the effect.

Multivariate analysis using a generalised linear model was performed to evaluate the association of AFC, heifer BW, year‐period and season, with the outcome variables of lactation performance. Data from 163 animals that had more than 200 days in milk during the first lactation were included in this analysis. The 305‐day milk yield and lifetime daily milk yield during the first lactation were the outcome variables. If a significant difference was detected, multiple comparisons were performed using a Tukey's HSD test to analyse the differences between the categories for each effect.

## RESULTS

### Descriptive analysis

**FIGURE 1:**
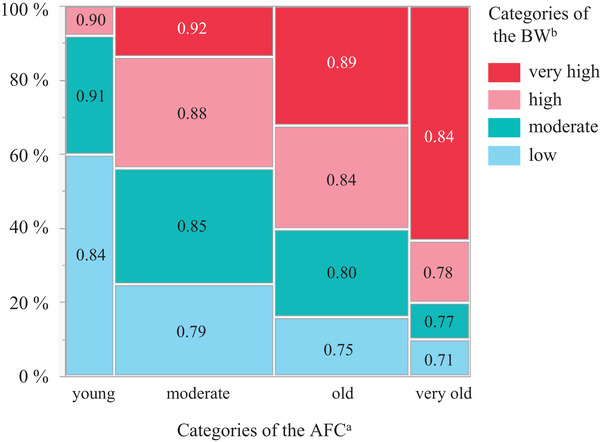
Mosaic diagram of the combination between the age at first calving (AFC)^a^, the bodyweight (BW)^b^ and the average daily liveweight gain (ADG) (g/day) for each combination. ^a^Young: <22.5 months; moderate: 22.5 to <24.0 months; old: 24.0 to <25.5 months; very old: ≥25.5 months of AFC. ^b^Low: ≤625 kg; moderate: 626–654 kg; high: 655–683 kg; very high: ≥684 kg.

Table 1 shows the mean, median, minimum and maximum values for the AFC and BW (heifer and calf) for each category. Table 2 presents the milk productivity and reproductive performance of heifers enrolled in the study. Figure 1 shows the mosaic diagram of the combination between the AFC and BW and the ADG, for each combination. The mean values for ADG were 872, 853, 834 and 810 g/day for young, moderate, old and very old AFC heifers, respectively. Sixty percent of heifers with less than 22.5 months AFC had low BW, while 63% out of heifers with more than or equal to 25.5 months AFC had very high BW at first calving.

**TABLE 1:** Descriptive analysis for the categories of the age at first calving (AFC) and bodyweight (BW) (heifer and calf) in this study.

Effects	Categories	*n*	Mean	Median	Minimum	Maximum
AFC (months)		203	24.03	23.90	20.84	28.67
	Young: <22.5	25	21.87	21.86	20.84	22.48
	Moderate: 22.5 to <24.0	80	23.29	23.30	22.52	23.93
	Old: 24.0 to <25.5	68	24.63	24.57	24.00	25.47
	Very old: ≥25.5	30	26.42	26.08	25.61	28.67
Heifers BW before the first calving (kg)		203	656.7	655.0	536.0	803.0
	Low: ≤625	49	600.7	604.0	536.0	625.0
	Moderate: 626–654	52	641.9	642.5	626.0	654.0
	High: 655–683	50	666.6	666.0	655.0	683.0
	Very high: ≥684	52	713.7	702.5	684.0	803.0
Delivered calf BW (kg)		203	41.8	41.5		75.5
	Low: 21.0–38.0	51	35.5	36.0	21.0	38.0
	Moderate: 38.5–44.0	101	41.2	41.5	38.5	44.0
	High: 44.5–75.5	51	49.0	46.5	44.5	75.5

**TABLE 2:** Milk productivity and reproductive performance of the heifers enrolled in the analysis

	Items	*n*	Mean	Median	Minimum	Maximum
Productive performance	Short lactation cows (%)	40 (18.5)				
	Age at the end of first lactation (months)	163	34.1	33.9	30.9	38.7
	305‐day milk yield (kg)	163	8069.5	8035.9	5590.5	10,949.4
	Lifetime daily milk yield (kg/day)	163	7.79	7.79	5.09	10.95
Reproductive performance	Heifers artificially inseminated (%)	172 (84.7)				
	The interval from calving to the first service (days)	172	85.6	82.5	45.0	179.0
	Pregnant cows at first service (%)	97 (56.4)				
	Total pregnant cows (%)	160 (93.0)				
	Days open (days)	160	107.4	94.0	45.0	296.0

### Calving difficulty and stillbirth

Table 3 shows the multinomial logistic regression analysis results for the effect of AFC, BW (heifer and calf), year‐period and season, contributing to the incidence of difficult calving and the prevalence of stillbirth. There was a significant association between the incidence of the calving difficulty and the prevalence of stillbirth with heifer BW (*p* < 0.05). The incidence of difficult calving was significantly higher in the animals with low BW compared with the heifers with moderate BW (odds ratio = 7.23, 95% CI: 1.514–34.59, *p* < 0.05). The prevalence of stillbirth was 11.4 and 5.4 times higher in the animals with low BW compared with the animals with moderate and high BW (95% CI: 2.032–216.0 and 1.299–36.77, respectively, *p* < 0.05).

**TABLE 3:** Multiple logistic regression analysis for the incidence of difficult calving (≥2 scores) and the prevalence of stillbirth

		Difficult calving		Stillbirth	
Effects	df	Likelihood ratio chi‐square	*p*‐Value	Likelihood ratio chi‐square	*p*‐Value
AFC^a^	3	2.42	0.49	2.80	0.42
Heifer BW^b^	3	8.21	0.04	11.1	0.01
Calf BW^c^	2	2.50	0.28	0.80	0.67
Year‐period^d^	3	1.49	0.68	4.04	0.26
Season^e^	3	6.11	0.11	2.43	0.49

Abbreviations: AFC, age at first calving; BW, bodyweight.

^a^ Young: <22.5 months; moderate: 22.5 to <24.0 months; old: 24.0 to <25.5 months; very old: ≥25.5 months of AFC.

^b^ Low: ≤625 kg; moderate: 626–654 kg; high: 655–683 kg; very high: ≥684 kg.

^c^ Low: 21.0–38.0 kg; moderate: 38.5–44.0 kg; high: 44.5–75.5 kg.

^d^ Year‐period 1:1999–2002; period 2: 2003–2006; period 3: 2007–2009; period 4: 2010–2012.

^e^ Spring: April–June; Summer: July–September; Autumn: October–December; Winter: January–March.

### Reproductive performance

The interval from calving to the first service was significantly associated with the year‐period (*p* < 0.01), but not AFC, BW (heifer and calf) and season. The lowest mean interval was recorded for the heifers calving in period 1 (70.4 ± 20.4 days), which was significantly different from animals calving in periods 2–4 (86.8 ± 23.5 days, 96.3 ± 20.9 days and 89.3 ± 22.9 days, respectively, *p* < 0.05). There was no effect detected for AFC, BW (heifer and calf), year‐period and season, on the number of days open.

### Lactation performance and calf production

No significant difference was detected between the effects of AFC, heifer BW, year‐period and season, on the frequency of the heifers with short lactation.

There was a significant association between 305‐day milk yield and year‐periods, while not with AFC, BW and season (Table 4). The lowest mean value of 305‐day milk yield was recorded for the heifers calving in period 1, which was significantly lower than heifers calving in period 3 (7683.3 ± 1032.8 kg vs. 8381.0 ± 1082.2 kg). An impact of the year‐period on the lifetime daily milk yield was observed (Table 4); the mean value was significantly higher for the heifers calving in periods 2 and 3, compared with those calving in period 1 (7.8 ± 0.8 kg and 8.2 ± 1.1 kg vs. 7.0 ± 1.9 kg). In addition, there was a significant association between the lifetime daily milk yield and AFC (Table 4); the highest mean value was recorded for the heifers in the young AFC group, which was significantly different from animals with the moderate and old AFC group (8.2 ± 1.0 kg vs. 7.8 ± 1.4 kg and 7.6 ± 1.5 kg). The BW before the first calving had a significant impact on the BW for delivered calves (Table 4); the higher mean value for the calf was detected for the heifers with very high BW compared with that with low and moderate (44.6 ± 6.6 kg vs. 40.5 ± 5.4 kg and 40.2 ± 6.6 kg).

**TABLE 4:** Results of the generalised linear model to evaluate the association of age at first calving (AFC), heifer bodyweight (BW), year‐period and season, with 305‐day milk yield (*p* = 0.0050), overall lifetime yield (*p* = 0.0033) and BW for delivered calves (*p* = 0.0462)

Outcomes	Effects	df	*F*‐value	*p*‐Value
305‐day milk yield	AFC^a^	3	0.4075	0.7479
	Heifer BW^b^	3	2.4068	0.0695
	Year‐period^c^	3	2.9213	0.0360
	Season^d^	3	1.7880	0.1519
Overall lifetime yield	AFC^a^	3	3.4998	0.0170
	Heifer BW^b^	3	0.7375	0.5312
	Year‐period^c^	3	3.5106	0.0168
	Season^d^	3	2.0367	0.1111
BW for delivered calves	AFC^a^	3	0.3061	0.8209
	Heifer BW^b^	3	3.0268	0.0314
	Year‐period^c^	3	1.5363	0.2074
	Season^d^	3	0.2399	0.8684

^a^ Young: <22.5 months; moderate: 22.5 to <24.0 months; old: 24.0 to <25.5 months; very old: ≥25.5 months of AFC.

^b^ Low: ≤625 kg; moderate: 626–654 kg; high: 655–683 kg; very high: ≥684 kg.

^c^ Year‐period 1: 1999–2002; period 2: 2003–2006; period 3: 2007–2009; period 4: 2010–2012.

^d^ Spring: April–June; Summer: July–September; Autumn: October–December; Winter: January–March.

## DISCUSSION

Our retrospective study indicated that the incidence of difficult calving and the prevalence of stillbirth were significantly associated with heifer BW and not with AFC. Specifically, low BW heifers had a higher incidence of calving problems compared with moderate BW heifers. Even so, the increase in calving problems had no adverse impact on subsequent reproductive performance. The 305‐day milk productivity in this study was not dependent on the AFC and BW at the first calving. Consequently, the overall lifetime yield during the first lactation was significantly higher in young AFC heifers compared to the moderate and old AFC heifers.

One study reported that heifers with early AFC and low BW (<23.0 months and <578 kg) had a higher incidence of calving difficulty, resulting in more stillborn compared with the older and heavier heifers (23.0–24.6 months and 579–613 kg).^10^ And further, the former had lower conception rates and longer days open than the latter.^10^ As well as being underweight, the over‐condition could impair the reproductive performance of replacement heifers. According to a large‐scale study in the UK, heifers calved more than 30 months (3.9 of body condition score [BCS]) suffered from the highest proportion of dead calves compared with heifers calved less than 23 months (2.1 of BCS) at first calving.^5^ In the previous study, the older AFC heifers had a lower first service conception rate compared with the younger heifers.^5^ In the present study, very high BW heifers calved significantly heavier calves than low and moderate BW heifers, while the incidence of difficult calving and prevalence of stillbirth was significantly higher in low BW heifers than in the greater BW. However, the increased calving problems had no adverse impact on reproductive performance.

These contradictory results might be caused by the difference in herd management levels between those studies; namely, appropriate calving assistance, at least at the experimental herd level, prevents calf losses and protects the subsequent reproductive performance of the early calving heifers. Increasing the size of dairy herds can place added pressure on existing labour and infrastructure, resulting in minimal/poor management of the replacement stock.^26^ Good management practices for replacement heifer calving could maximise the benefits of accelerating AFC; in contrast, poor management could emphasise the disadvantages of reducing AFC.

Accelerated body growth during the prepubertal period may have a negative effect on milk productivity. One study compared milk productivity between heifers with accelerated body growth and those without (ADG, 0.93 kg/day vs. 0.78 kg/day; AFC, 21.7 months vs. 24.6 months).^11^ The results indicated that the acceleration was associated with reducing milk components, milk fat and protein, but not with the amount of milk yield during the first lactation.^11^ Another study investigated the effect of three dietary energy treatments on milk productivity (ADG, 0.68 kg/day vs. 0.83 kg/day vs. 0.94 kg/day; AFC, 24.5 months vs. 22.0 months vs. 21.3 months, respectively).^9^ Reductions in the actual 305‐day and 4% fat‐corrected milk yields were reported in the heifers managed with the highest energy ration compared to that with the lowest (9387 kg vs. 9873 kg and 8558 kg vs. 9008 kg, respectively).

Similarly, these harmful effects were reported by retrospective studies. In one study, using 1905 calving records, milk yield was examined in relation to the first lactation according to AFC designated as low, medium and late, respectively (AFC, <23.0, 23.0–24.6 and >24.6 months; BW, 570.9, 603.3 and 650.4 kg at calving, respectively).^10^ Milk production in early lactation was similar among the three groups; although after 50 days, in milk heifers in the low group produced less milk than those in the medium and late groups, resulting in lower milk production for low AFC compared to medium and late (33.4 kg/day vs. 34.4 and 34.7 kg/day, respectively). On the contrary, another study analysed a dataset of 445 heifers retrospectively, which indicated that early calving (<23.0 months; ADG, 0.80–0.85 kg/day) was not correlated with reducing the amount of first milk lactation compared with that in older calving heifers.^5^

In our retrospective study, the 305‐day milk yield significantly increased year by year, which may be a result of 13 years of genetic improvement in milk productivity in this experimental herd. During this period, the AFC and BW before the first calving were not significantly associated with the 305‐day milk productivity. Consequently, young AFC heifers (AFC, <22.5 months; ADG, 0.87 kg/day) had significantly higher lifetime daily milk yield during the first lactation compared to older heifers. This result was in keeping with a previous report for this herd; namely, the early calving heifers (AFC, 21.5 months; ADG, 0.88 kg/day) had no negative effects on first lactation performance.^8^

The effect of AFC on lactation performance was not always consistent; the retrospective analysis must include many confounders, making it difficult to interpret the effect of AFC correctly. Previous studies focusing on the effect of AFC could not consider the effect of BW simultaneously because no information was available on a population basis.^18^, ^19^, ^20^, ^22^ The present analysis, including the effect of AFC, BW, genetic changes and calving season, on the performance of the first lactation, indicated that heifers calving at less than 22.5 months had significantly higher daily milk yield until the end of the first lactation than heifers calved after that. This is an important observation because dairy producers often assume that early breeding causes severe disadvantages during the first lactation period.

According to the estimation of the cost of a heifer produced in‐house, using data from Japanese commercial dairy herds, the average rearing cost was 386 yen (2.38 GBP) per day.^27^ In the present study, young AFC heifers conceived earlier, by approximately 2.8 and 4.6 months, compared with the old and very old AFC heifers; therefore, it is expected that the reduction of the AFC from more than 24 to 22 months could reduce the rearing cost by approximately 32,000 to 53,000 yen (197–327 GBP) in this experimental herd. The reduction of AFC could save replacement costs. However, it cannot be ignored that there are various effects on dairy profitability in addition to the AFC. For instance, nutritional management, differences in colostrum intake and/or growth rate to weaning, neonatal disease, body condition and postpartum disease, which was not included in our analysis. By investigating these effects simultaneously, it will be possible to clarify the effect of the AFC on dairy profitability.

## AUTHOR CONTRIBUTIONS

Hiromi Kusaka and Minoru Sakaguchi were responsible for the conception and design. Minoru Sakaguchi and Takeshi Yamazaki were responsible for the acquisition of data. Hiromi Kusaka carried out the analysis. Hiromi Kusaka, Minoru Sakaguchi and Takeshi Yamazaki were responsible for interpreting the results, drafting the manuscript and giving final approval of the version to be published.

## CONFLICTS OF INTEREST

The authors declare they have no conflicts of interest.

## FUNDING INFORMATION

The authors have not declared a specific grant for this research from any funding agency in the public, commercial or not‐for‐profit sectors.

## ETHICS STATEMENT

The Animal Care and Use Committee of the NARO approved the experimental protocol.

## Data Availability

Data available on request from the authors.
